# Invasive Pneumococcal Disease among Childbearing-Age Women, United States, 2007–2023

**DOI:** 10.3201/eid3202.251279

**Published:** 2026-02

**Authors:** Namrata Prasad, Sopio Chochua, Bridget J. Anderson, Kathy M. Angeles, Meghan Barnes, Lee H. Harrison, Corinne Holtzman, Jessica R. Howard-Anderson, Shannon O’Brien, Susan Petit, Arthur Reingold, William Schaffner, Lesley McGee, Adam L. Cohen, Miwako Kobayashi

**Affiliations:** Centers for Disease Control and Prevention, Atlanta, Georgia, USA (N. Prasad, S. Chochua, L. McGee, A.L. Cohen, M. Kobayashi); New York State Department of Health, Albany, New York, USA (B.J. Anderson); New Mexico Department of Health, Santa Fe, New Mexico, USA (K.M. Angeles); Colorado Department of Public Health and the Environment, Denver, Colorado, USA (M. Barnes); Johns Hopkins Bloomberg School of Public Health, Baltimore, Maryland, USA (L.H. Harrison); Minnesota Department of Health, St. Paul, Minnesota, USA (C. Holtzman); Emory University School of Medicine, Atlanta (J.R. Howard-Anderson); Oregon Health Authority, Portland, Oregon, USA (S. O'Brien); Connecticut Department of Public Health, Hartford, Connecticut, USA (S. Petit); California Emerging Infections Program, Oakland, California, USA (A. Reingold); University of California, Berkeley, California (A. Reingold); Vanderbilt University School of Medicine, Nashville, Tennessee, USA (W. Schaffner)

**Keywords:** pneumococcal infections, Streptococcus pneumoniae, bacteria, pregnancy, vaccines, United States

## Abstract

US data on invasive pneumococcal disease incidence among pregnant and postpartum women are limited. We estimated incidence in those groups using population-based surveillance. Compared with nonpregnant women of childbearing age, incidence was similar for pregnant women but 3.5 times higher for postpartum women. Our findings could inform pneumococcal vaccine recommendations.

*Streptococcus pneumoniae* is a leading cause of serious bacterial infections, including invasive pneumococcal disease (IPD), defined as *S. pneumoniae* infection in a normally sterile site (e.g., blood, cerebrospinal fluid). IPD most commonly affects young children, older adults, and people with certain underlying conditions (*1*). For adolescents and adults <50 years of age with specific conditions, the Advisory Committee on Immunization Practices (ACIP) recommends use of 15-valent, 20-valent, or 21-valent pneumococcal conjugate vaccines (PCV) (*2*). However, ACIP has not reviewed data on PCV use in pregnant or postpartum women and does not offer recommendations for those groups (*3*). The American College of Obstetricians and Gynecologists, by contrast, recommends PCV for pregnant women at increased risk for severe disease (*4*). To help address this gap in recommendations, we analyzed IPD epidemiology among US women of childbearing age. We conducted research in accordance with applicable federal law and Centers for Disease Control and Prevention policy.

We included all IPD cases among pregnant, postpartum, and nonpregnant childbearing-age women (15–44 years of age) reported by Active Bacterial Core surveillance (ABCs) during 2007–2023. ABCs is an active laboratory- and population-based, multistate surveillance system. We defined IPD as isolation of *S. pneumoniae* bacteria from a sterile site in a surveillance-area resident (*5*). We defined the postpartum period as <30 days after delivery.

We reported IPD incidence as cases per 1,000 person-years. We estimated denominators for incidence rates using published methods (*6*). In brief, we estimated the number of pregnant women by multiplying annual live births, induced abortions, and early and late fetal losses by the mean proportion of the year a woman is pregnant for each outcome (live birth = 0.75, abortion = 0.12, early loss = 0.14, late loss = 0.52). We estimated the number of postpartum women by multiplying live births by one twelfth. We estimated the number of nonpregnant women by subtracting pregnant and postpartum women from the total number of childbearing-age women in ABCs areas. We used a mixed-effects Poisson model, including ABCs site as a random effect, to estimate incidence rates, incidence rate ratios (IRRs), and 95% CIs.

We compared demographic and clinical characteristics and PCV20 and PCV21 serotype groups by pregnancy status across cases. We assessed differences using *t* test, Pearson χ^2^ test, or Fisher exact test and considered p<0.05 statistically significant. We conducted analyses using R version 4.0.4 (The R Project for Statistical Computing, https://www.r-project.org) across the entire surveillance period and during the more recent period of 2019–2023 to account for changes in PCV recommendations and serotype distribution over time.

During 2007–2023, we identified 3,651 IPD cases among childbearing-age women, including 146 (4.0%) pregnant, 61 (1.7%) postpartum, and 3,444 (94.4%) nonpregnant women (Figure). Gestational week data were available for 77 (52.7%) cases; of those, 12 (15.6%) were in the first trimester, 31 (40.3%) the second trimester, and 34 (44.2%) the third trimester at the time of illness. During 2019–2023, IPD incidence in pregnant women (0.017 [95% CI 0.011–0.028] per 1,000 person-years) was not significantly different from that in nonpregnant women (0.025 [95% CI 0.019–0.034] per 1,000 person-years) (IRR 0.68 [95% CI 0.47–1.00]). In contrast, postpartum women (0.088 [95% CI 0.048–0.161] per 1,000 person-years) had higher IPD risk than did nonpregnant women (IRR 3.49 [95% CI 2.06–5.90]).

**Figure:**
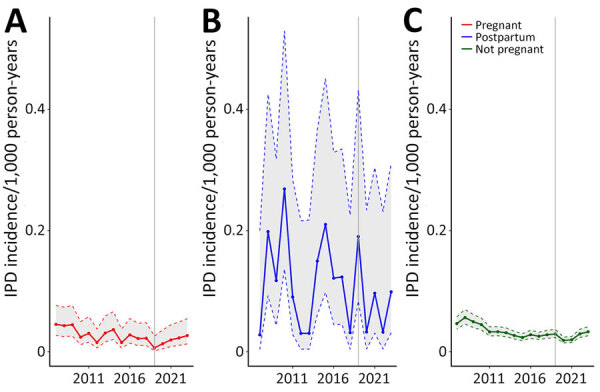
Incidence of IPD among pregnant (A), postpartum (B), and nonpregnant (C) women of childbearing age, United States, 2007–2023. Data were obtained from Active Bacterial Core surveillance. Shaded areas represent 95% CIs. IPD, invasive pneumococcal disease.

Compared with IPD cases among nonpregnant women (mean age 35 years), pregnant (mean age 29 years; p<0.001) and postpartum case-patients (mean age 30 years; p<0.001) were younger and less likely to have underlying conditions (pregnant women = 45.2%; postpartum women = 41.0%; nonpregnant women = 69.9%; p<0.001) (Table). That difference was particularly noticeable for women with immunocompromising conditions. Pregnant women had a lower IPD case-fatality ratio (2.1%) than did nonpregnant women (7.9%; p = 0.014). Among IPD cases in pregnant and postpartum women, most (>75%) experienced favorable fetal outcomes (no pregnancy loss or clinical infection). During 2019–2023, the proportion of IPD cases caused by PCV20, PCV21, or nonvaccine serotypes did not vary significantly by pregnancy status; 484/778 (62.2%) of cases were caused by serotypes covered by PCV20 and 587/778 (75.4%) by serotypes covered by PCV21 (Table).

**Table:** Characteristics of invasive pneumococcal disease cases among pregnant, postpartum, and nonpregnant childbearing-age women, United States, 2007–2023*

Characteristic	Pregnant, n = 146			Postpartum, n = 61		Nonpregnant, n = 3,444
	Value	p value		Value	p value	
Mean age, y (IQR)	29 (25–34)	<0.001		30 (25–34)	<0.001	35 (30–41)
Clinical manifestations, no. (%)†						
Bacteremia	33 (22.6)	0.012		10 (16.4)	0.850	506 (14.7)
Meningitis	7 (4.8)	0.135		5 (8.2)	1.000	299 (8.7)
Pneumonia	93 (63.7)	0.142		38 (62.3)	0.263	2,403 (69.8)
Other	25 (17.1)	0.008		19 (31.1)	0.626	947 (27.5)
Patient outcome						
Admitted to ICU	28/89 (31.4)	0.056		15/47 (31.9)	0.205	1,000/2,369 (42.2)
Death	3/146 (2.1)	0.014		0/61 (0.0)	NA	273/3,443 (7.9)
Fetal outcome in cases with available data						
Survived, no apparent illness or still pregnant	98/118 (83.1)			42/55 (76.4)		NA
Survived, clinical infection	1/118 (0.8)			3/55 (5.5)		NA
Miscarriage/stillbirth	18/118 (15.3)			8/55 (14.5)		NA
Underlying conditions, no. (%)‡	66 (45.2)	<0.001		25 (41.0)	<0.001	2,407 (69.9)
Chronic conditions	53 (36.3)	0.463		20 (32.8)	0.336	1,367 (39.7)
Immunocompromising conditions, cerebrospinal fluid leak, or cochlear implant	13 (8.9)	<0.001		5 (8.2)	<0.001	1,040 (30.2)
Healthy	80 (54.8)	<0.001		36 (59.0)	<0.001	1,037 (30.1)
Serotyped IPD cases during 2019–2023						
PCV20-covered serotypes§	16/23 (69.6)	0.598		8/12 (66.7)	1.000	460/743 (61.9)
PCV21-covered serotypes¶	15/23 (65.2)	0.346		8/12 (66.7)	0.497	564/743 (76.0)
Covered by neither	1/23 (4.3)	1.000		2/12 (16.7)	0.265	61/743 (8.9)

*Values are no. positive/total no. (%) except as indicated. ICU, intensive care unit; IPD, invasive pneumococcal disease; IQR, interquartile range; NA, not applicable; PCV20, 20-valent pneumococcal conjugate vaccine; PCV21, 21-valent pneumococcal conjugate vaccine. †Sum of proportions could be >100% because case-patients could have had >1 clinical manifestation identified. ‡Conditions included chronic conditions (alcoholism; chronic heart, liver, or lung disease; chronic renal failure; cigarette smoking; diabetes mellitus) and immunocompromising conditions (congenital or acquired asplenia; generalized malignancy; HIV; Hodgkin disease; immunodeficiency; iatrogenic immunosuppression; leukemia, lymphoma, or multiple myeloma; nephrotic syndrome; solid organ transplant; or sickle cell disease or other hemoglobinopathies). Cerebrospinal fluid leak and cochlear implant were grouped together with immunocompromising conditions to align with 2023 vaccine recommendations (*3*). §PCV20 serotypes: 1, 3, 4, 5, 6A, 6B, 7F, 8, 9V, 10A, 11A, 12F, 14, 15B, 18C, 19A, 19F, 22F, 23F, and 33F. ¶PCV21 serotypes: 3, 6A, 7F, 8, 9N, 10A, 11A, 12F, 15A, 15B, 15C, 16F, 17F, 19A, 20A, 22F, 23A, 23B, 24F, 31, 33F, and 35B. PCV21 is approved for the prevention of invasive pneumococcal disease caused by serotype 15B based upon prespecified criteria for the proportion of participants with 4-fold or more rise in opsonophagocytic activity responses. Source: US Food and Drug Administration (https://www.fda.gov/media/179426/download?attachment).

Our findings align with previous studies showing no increased IPD risk during pregnancy but elevated risk postpartum (*7*,*8*). One study also found IPD risk was slightly increased in third-trimester pregnant women compared with nonpregnant women (*7*). Similar stratifications were not possible in our study because we lacked complete gestational week data. In our study, pregnant and postpartum women were younger and healthier than nonpregnant women, which likely reflected the underlying population that becomes pregnant. Those differences might have influenced observed IPD risk and outcomes; we could not account for them in incidence and IRR estimations because we lacked denominator data stratified by age and underlying conditions. Further studies are needed to determine factors associated with increased risk for IPD in the postpartum period.

In conclusion, we found that IPD incidence was similar among pregnant and nonpregnant women but higher among postpartum women. Our findings could inform pneumococcal vaccine recommendations for women of childbearing age. 
